# The Role Played by Ionic Liquids in Carbohydrates Conversion into 5-Hydroxymethylfurfural: A Recent Overview

**DOI:** 10.3390/molecules27072210

**Published:** 2022-03-29

**Authors:** Salvatore Marullo, Francesca D’Anna

**Affiliations:** Dipartimento STEBICEF, Università degli Studi di Palermo, Viale delle Scienze Ed. 17, 90128 Palermo, Italy; salvatore.marullo@unipa.it

**Keywords:** ionic liquids, carbohydrates conversion, 5-hydrodroxymethylfurfural, homogenous catalysis, heterogenous catalysis

## Abstract

Obtaining industrially relevant products from abundant, cheap, renewable, and low-impacting sources such as lignocellulosic biomass, is a key step in reducing consumption of raw fossil materials and, consequently, the environmental footprint of such processes. In this regard, a molecule that is similar to 5-hydroxymethylfurfural (5-HMF) plays a pivotal role, since it can be produced from lignocellulosic biomass and gives synthetic access to a broad range of industrially important products and polymers. Recently, ionic liquids (ILs) have emerged as suitable solvents for the conversion of biomass and carbohydrates into 5-HMF. Herein, we provide a bird’s-eye view on recent achievements about the use of ILs for the obtainment of 5-HMF, covering works that were published over the last five years. In particular, we first examine reactions involving homogeneous catalysis as well as task-specific ionic liquids. Then, an overview of the literature addressing the use of heterogeneous catalysts, including enzymes, is presented. Whenever possible, the role of ILs and catalysts driving the formation of 5-HMF is discussed, also comparing with the same reactions that are performed in conventional solvents.

## 1. Introduction

During the last decades, the high standards of living of the modern society has induced a continuous growth in energy demand and a parallel increasing consumption in fossil fuels [1,2]. On one side, this has caused a significant impact of the anthropogenic activities in the environment, as accounted for by the widespread environmental degradation that affects land, air, and water [3]. On the other hand, a significant raise in the price of fossil fuel sources has been also detected. The above scenario explains why, in the last few years, one of the main aims of research activity has been the strengthening in using renewable sources and, in particular, biomasses i.e., organic matter having recent origin and that are renewable at a relatively high rate [4,5].

In this context, the use of lignocellulosic biomass plays a significant role [6]. Indeed, it is one of the most abundant sources on the earth, deriving from agricultural waste, but also from foods, animal, and human waste. Furthermore, differently from other renewable sources, it is not involved in the food chain, avoiding competitive interests, and it is of low cost [7,8].

**Scheme 1:**
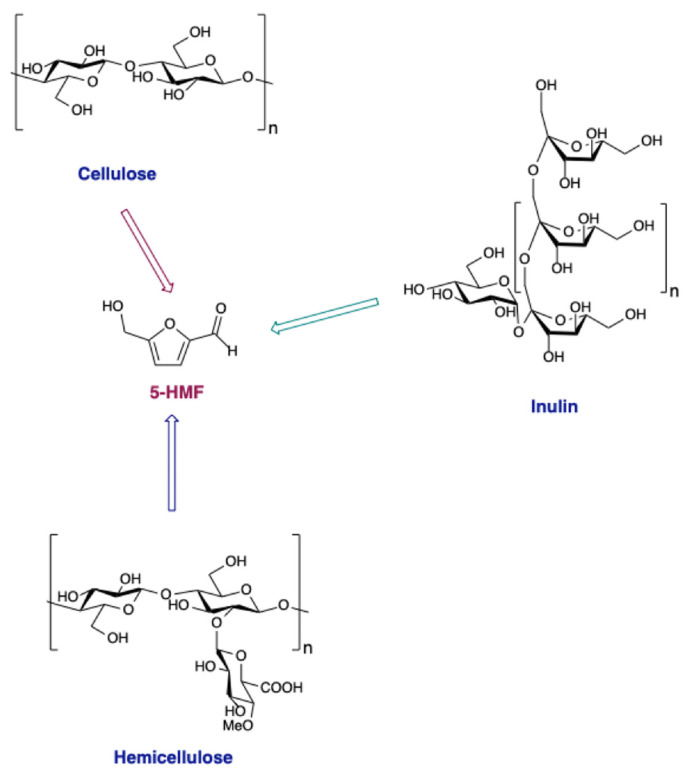
Representative natural polymers from which 5-HMF can be obtained.

Lignocellulosic biomass is highly heterogeneous as it comprises of cellulose, hemicellulose, and lignin, besides fats, proteins, free sugars, and so on. However, among the above components, cellulose and hemicellulose have proved to be highly important as, after saccharification, they can be used to obtain very important chemical platform, such as 5-hydroxymethylfurfural (5-HMF) (Scheme 1). This can be obtained from the acid catalyzed dehydration of glucose, fructose, galactose, xylose, arabinose, ribose, lactose, sucrose, and maltose. 

**Scheme 2:**
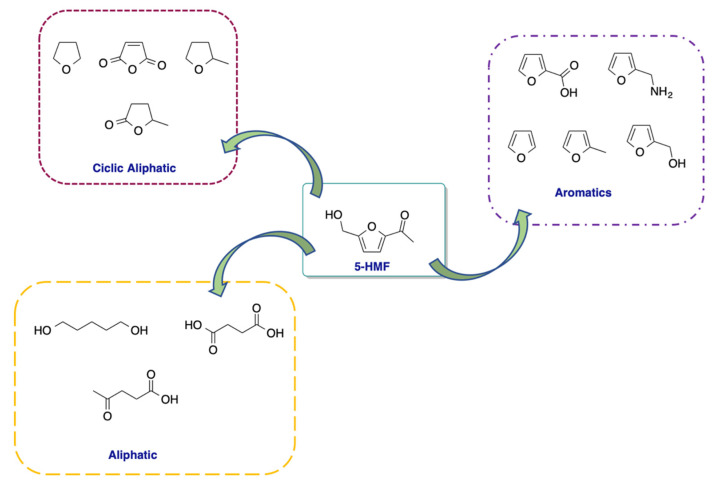
Structure of products of industrial values that can be obtained from 5-HMF.

It can be considered as a multifunctional molecule due to the presence of an alcoholic and aldehydic group, together with the aromatic furan ring. This allows its transformation to more than 80 high-value added compounds and explains the reason why it is called the “sleeping giant” in the field of chemistry concerning raw materials (Scheme 2) [9,10].

5-HMF was firstly isolated, in a lab scale, in 1831 and the first facility for its production on a large scale started in 1921 [11,12]. Currently, the worldwide annual production amounts to 652 kilotons. However, the large part of such technologies shows several issues, such as the use of highly corrosive acidic catalysts that pose limitations from an industrial point of view; the use of large amount of energy, above all as far as the dehydration of glucose is concerned; and the high tendency of 5-HMF to give secondary processes and polymerization reactions [13,14]. Notwithstanding the above issues, a lot of efforts have been carried out to optimize reaction conditions for the obtainment of 5-HMF, both in water and in conventional organic solvents. However, in the last decades, also the use of ionic liquids (ILs) has roused a surge of interest [15,16,17].

**Scheme 3:**
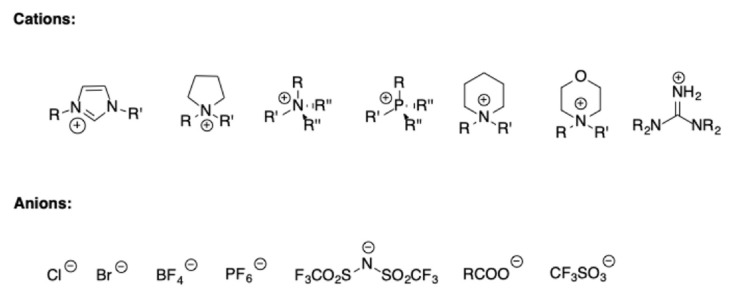
Structure of the most common IL cations and anions.

As it is well known, ILs are organic salts having melting point lower than 100 °C. Generally, they are featured by the presence of organic cations, whereas the anions can be both organic or inorganic in nature (Scheme 3).

Frequently, they are liquid at room temperature and can be used as solvents. They show low vapor pressure and flammability, that indicate them as benign alternative to conventional organic solvents.

ILs have been successfully applied to perform carbohydrates conversion into 5-HMF. Indeed, with respect to conventional solvents, they allow the achievement of higher yields, using milder conditions. Furthermore, their low miscibility with conventional solvents also drives the recovery of the target product by liquid–liquid extraction, frequently allowing the recycling of the solvent/catalyst system.

A strong point in the use of ILs in such processes also lies in the possibility of tailoring the features of ILs for the target applications. This can be performed by anchoring catalytic functions on the cation or anion structure, awarding the IL of the dual role of solvent and catalyst, obtaining the so-called task specific ILs (TSILs).

The wide interest in the application of ILs in this field is testified by the large number of papers that have been published since 2003 (more than 450) and some interesting reviews have also analysed the topic [18,19,20,21,22]. This is the reason why we focused our attention in the most recent results, covering the last five-year period, and addressing the use of ILs as solvents for both homogeneous and heterogeneous catalysis, together with the applications of TSILs.

## 2. Catalysis in Homogeneous Phase

The obtainment of 5-HMF from monosaccharides such as glucose and fructose has been widely investigated in homogeneous conditions, by using both solvents and catalysts of different nature. As far as the use of ILs as solvents is concerned, different reports highlight the very good performance that is detected in halide-based ILs [15,23,24]. The high solvent activity, in these cases, is mainly ascribed to the high anion coordination ability of the IL anion that warrants high dissolution ability towards carbohydrates thanks to the establishment of a hydrogen bond with hydroxyl groups of carbohydrates. In general, less satisfactory results have been obtained in ILs featured by the presence of less coordinating anions such as [BF_4_^−^], [PF_6_^−^] and [NTf_2_^−^] and so on.

Notwithstanding the success in using halide-based ILs, in terms of both conversion and yield, serious concerns have been detected in the 5-HMF separation and paradoxically, this is the result of the hydrogen bond interaction between the hydroxyl group in the 5-HMF and the IL anion. In light of the above consideration, efforts have been carried out with the aim to improve carbohydrates dehydration in ILs featured by the presence of non-coordinating anions. 

**Scheme 4:**
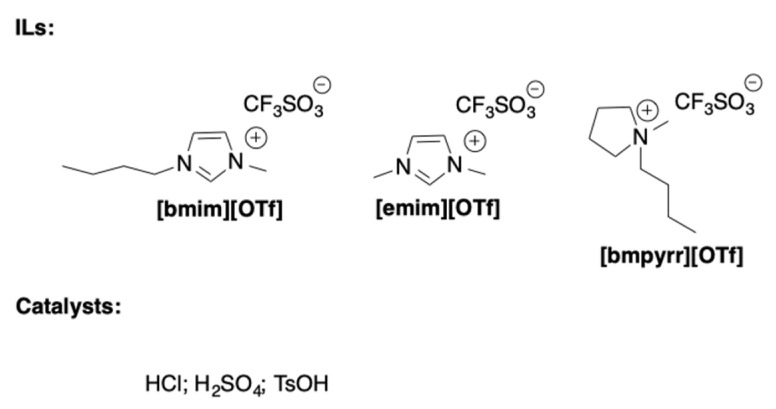
Structure of non-coordinating anion based ILs and acidic catalysts.

To this aim, a very interesting report investigated the possibility to perform acid catalyzed fructose dehydration in [OTf^-^]-based ILs [25]. In particular, solvents differing for the cation nature were used (Scheme 4) and the effect of different operational parameters such as the nature of the acidic catalyst, the amount of water in the reaction mixture, the fructose loading, and the reaction temperature were evaluated.

The data that were collected demonstrated that, at 90 °C, among the tested catalysts, the best performance was detected by using HCl in the presence of 3.5% of water. In the above experimental conditions, using a 14% of fructose loading, allowed the obtainment of a 5-HMF yield that was equal to 74% in 10 min. The above result was ascribed to the good stability of 5-HMF towards self-condensation side reactions in the used IL/water mixture.

As far as the temperature and fructose loading are concerned, the data that were reported demonstrated that a gradual increase in the substrate loading from 2 up to 14% induced a corresponding decrease in 5-HMF yield from 86% down to 72%. On the other hand, at 10% of fructose loading, the kinetic investigation that was performed in the temperature range 80–100 °C highlighted the opportunity to operate at 100 °C to obtain a 5-HMF yield equal to 81%, after 6 min. No significant effects were obtained by changing the nature of IL, accounting for a marginal effect deriving from the interaction between fructose and the IL cation at the high operational temperature. Differently, according to the starting hypothesis, the use of ILs having a non-coordinating anion, in the presence of a very small amount of water, significantly simplifies the 5-HMF extraction in diethyl ether (71%), opening the possibility of reusing the solvent.

The possibility of using hydrophobic ILs in the carbohydrates conversion into 5-HMF has been also performed by using [C_10_mim][OTf], as solvent system and HCl as an acidic catalyst [26]. The process was carried out taking into consideration fructose, glucose, and sucrose in order to test the efficiency of the catalytic system. The data that were reported evidence the high dependence of the experimental conditions from the nature of the carbohydrate. Less harsh experimental conditions were used in the case of fructose, that allowed the obtainment of 90% of yield in 5-HMF, after 60 min at 120 °C. Differently, in the case of glucose and sucrose, the process was carried out at 140 and 110 °C and gave rise to the highest yield in 5-HMF (20 and 80%) after 120 min. 

The great advantage in using such a kind of system remains in the possibility to recover 5-HMF by simply adding water, thanks to the high hydrophobicity of the solvent system. This allowed the reuse of the IL for at least three cycles, without loss in performance.

**Scheme 5:**
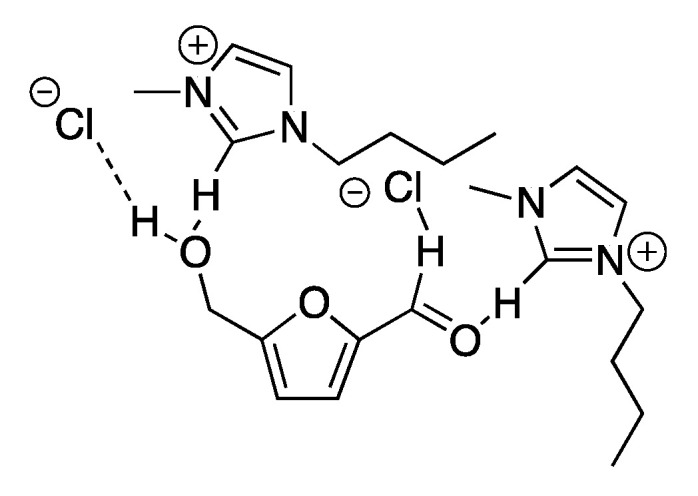
Structure of the complex [bmim][Cl]-5-HMF.

IL/water binary mixtures have been also used to carry out fructose, glucose, sucrose, cellobiose, inulin, and starch conversion into 5-HMF, by using metal sulfates as catalysts [27]. Optimization of the experimental conditions was initially performed by using fructose as a substrate (5 wt%), [bmim][Cl]/H_2_O (15 wt%) and different sulfate catalysts, at 140 °C for 1.5 h. The data that were collected evidence the highest efficiency of Fe(SO_4_)_2_ that was used for further experiments, aimed at the optimization of reaction time and temperature and [bmim][Cl] dosage. Under the best experimental conditions (140 °C, 1.5 h, [bmim]Cl = 15 wt%), a 5-HMF yield of 51.7% was obtained. In particular, the obtainment of 5-HMF and its stability in the reaction environment proved to be affected by the amount of [bmim][Cl]. The results of ^1^H-NMR and IR investigation allowed to foresee the formation of a [bmim][Cl-5-HMF complex, that was able to stabilize the reaction product towards both the humins formation and hydrolysis to lactic and formic acid (Scheme 5).

The proposed catalytic system was also efficient in the case of other biomass carbohydrates. However, higher reaction temperatures or longer reaction times were required to have a 5-HMF yield increase from 30 up to 40%. Interestingly, the proposed catalytic system also worked in water/THF (1:3; *v*/*v*) binary system. In this case, the continuous removal of 5-HMF in the organic phase allowed to drive the yield up to 65.8%.

**Scheme 6:**
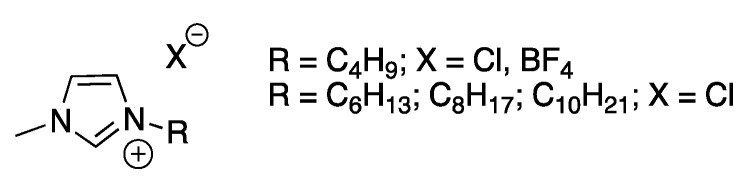
Structures of ILs used in combination with MoCl_3_.

In order to pay attention to the possibility of saving energy in processes that are aimed at carbohydrates conversion, the effect of microwaves irradiation on the glucose dehydration has been also investigated in [bmim][Cl] solution, using MoCl_3_ as a catalyst [28]. The process was also carried out under thermal heating by using 1-alkyl-3-methylimidazolium chlorides ILs, differing for the length of the alkyl chain. Furthermore, with the cation being the same [bmim^+^], the process was also performed using a less coordinating anion, such as [BF_4_^−^] (Scheme 6).

The first investigation that was carried out at 120 °C for 2.5 h, shed light on the effect of the nature of the IL, pointing out the negative effect of the alkyl chain elongation, that caused a decrease in 5-HMF yield from 18.39% ([bmim][Cl]) down to 7.65% ([dmim][Cl]). A similar drop in yield was also observed going from [bmim][Cl] (18.39%) to [bmim][BF_4_] (5.88%).

Important operational parameters, such as the reaction time and catalyst dosage were optimized under thermal conditions, allowing to identify as best reaction conditions: 120 °C, 2.5 h, 10 mol% of MoCl_3_. Microwaves irradiation, under 300 W at 120 °C, allowed the obtainment of higher 5-HMF yield (26.76%) after 5 min. Interestingly, the same protocol could be applied to different substrates, such as fructose and microcrystalline cellulose, obtaining 5-HMF yields that were equal to 48.2 and 11.5%, respectively.

Investigation of the reaction mechanism, on the grounds of a combined approach of ^13^C-NMR and tandem MS, evidenced the role that was played by the MoCl_3_/[bmim][Cl] system, that involved the glucose-fructose isomerization via the complexation of the open chain glucose and the consequent fructose dehydration, driven by the same catalytic system.

In a further attempt, the [bmim][Cl]/CrCl_3_ catalyzed dehydration of glucose was firstly analysed by using response surface methodology (RSM) [29]. The regression analysis was carried out under certain applied constraints, such as the catalyst amount, reaction temperature, and time, to maximize the 5-HMF yield. The optimum reaction conditions, determined as 125 °C for 120 min, in the presence of the 8 wt% of CrCl_3_, would give 65.6% of 5-HMF yield. The application of the above conditions gave 61.8% of 5-HMF yield which was in scope of 95% prediction interval. The process was also performed in the temperature range 90–120 °C that allowed estimating an activation energy that was equal to 120 kJ/mol. Interestingly, experimental procedure was also optimized in order to separate humins, that would have potential application as agricultural fertilizers, and recovery 5-HMF. In particular, in the last case, a nanofiltration separation process was carried out, allowing the recovery of 94.9% of 5-HMF that was present in the mixture and of IL/CrCl_3_ system, that can be reused for two further cycles.

**Scheme 7:**
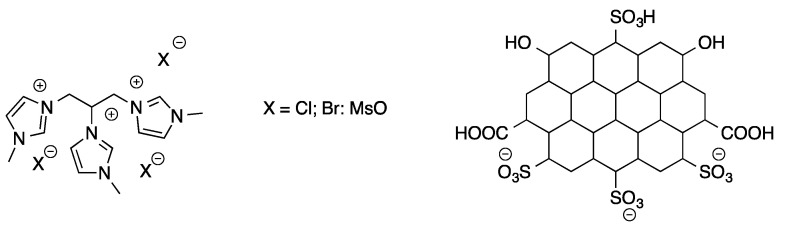
Structure of the tricationic ILs and CCSO_3_H co-catalyst.

The formation of 5-HMF from mono- and disaccharides, such as fructose, glucose, and sucrose was also studied in the presence of tricationic imidazolium salts [30]. With the aim to control the environmental impact of the process, tricationic salts featured by the presence of a glycerol spacer were used. Different anions, such as mesylate, hexafluorphosphate, chloride, and bromide were used (Scheme 7).

The process was initially performed in the temperature range 80–160 °C, for 3 h, using both neat ILs and their solution in THF, IPA, H_2_O/THF, and H_2_O/IPA.

Data that were collected evidenced the higher activity of the ILs on the pure form. Furthermore, among the tested anions, the mesylate proved the most efficient. Different reaction temperatures were selected as a function of the different nature of the substrate. In particular, fructose and sucrose gave the best yield in 5-HMF (93 and 72%) at 120 °C. In the case of glucose, the reaction that was performed at 140 °C, gave 51% of 5-HMF yield.

Interestingly, the catalytic activity of the less reactive ILs (Cl^−^, Br^−^ and PF_6_^−^ derivatives) was improved in the presence of the CCSO_3_H catalyst. The IL/co-catalyst combination induced a significant increase in 5-HMF yield. This was ascribed to the slow release of H^+^ from the co-catalyst that governs the initial rate of sugar dehydration. The proposed systems show a good recyclability. Both pristine and IL/co-catalyst systems were recycled for at least four times without a significant loss in performance.

**Scheme 8:**
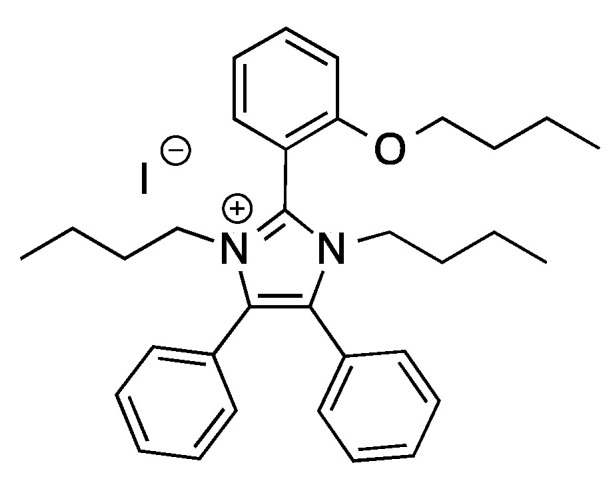
Structure of [DBDIm]I used as solvent for glucose dehydration.

Glucose dehydration has been also performed using ILs that are environmentally friendly, non-toxic, and easy to be produced on industrial scales, such as 1,3-dibutyl-2-(2-butoxyphenyl)-4,5-diphenylimidazolium iodide [DBDIm]I (Scheme 8) [31].

In this case, H_2_SO_4_ was used as Brønsted acidic catalyst and the optimization of experimental conditions allowed the obtainment of a 5-HMF yield that was equal to 82.2%, working at 100 °C, for 120 min. Thanks to the hydrophobic nature of the IL, 5-HMF was easily recovered by liquid–liquid extraction, using a toluene/water mixture (4:1; *v*/*v*) and the solvent was reused for five cycles. A detailed mechanistic picture was provided, showing the synergic action of H_2_SO_4_ and iodide anion in catalyzing the target process.

## 3. Catalysis in Homogeneous Phase and in the Presence of Task-Specific Ionic Liquids

**Scheme 9:**
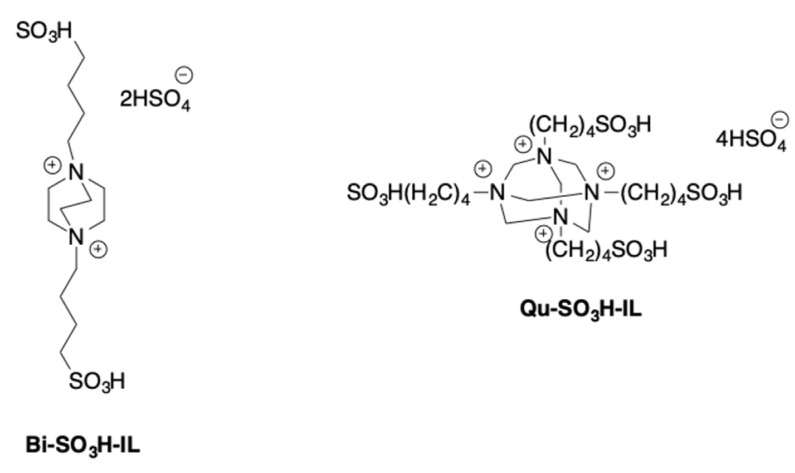
TSILs that were used for the fructose dehydration in SC/Sub-CO_2_/[bmim][Cl] system.

Given the acid catalyzed nature of the mechanism of sugar dehydration to 5-HMF, several efforts have been carried out in order to have the solvent and catalyst function in the same system. During the years, this has boosted the application of task-specific ILs (TSILs) in the above processes. In this context, fructose dehydration to 5-HMF has also been performed in the presence of TSILs, in SC/Sub-CO_2_/[bmim]Cl] as a solvent system [32]. In particular, TSILs based on triethylenediamine and hexamethylenetetramine nucleus were used as acidic catalysts (Scheme 9).

The data that were collected show that the yield in 5-HMF significantly increases with the increase in both pressure and reaction temperature, with the best results collected at 9 MPa and 120 °C, that in 0.5 h gave rise to the obtainment of 87.2% of 5-HMF. Longer reaction times induced a decrease in the 5-HMF yield, probably accounting for the polymerization and hydrolysis of 5-HMF. The use of [bmim][Cl], in combination with SC/Suc-CO_2_, proved to be a suitable combination thanks to the high solubility of compressed CO_2_ in ILs solution, that induces a decrease in the viscosity and facilitates the fructose dissolution in the reaction media, improving the reaction efficiency.

**Scheme 10:**
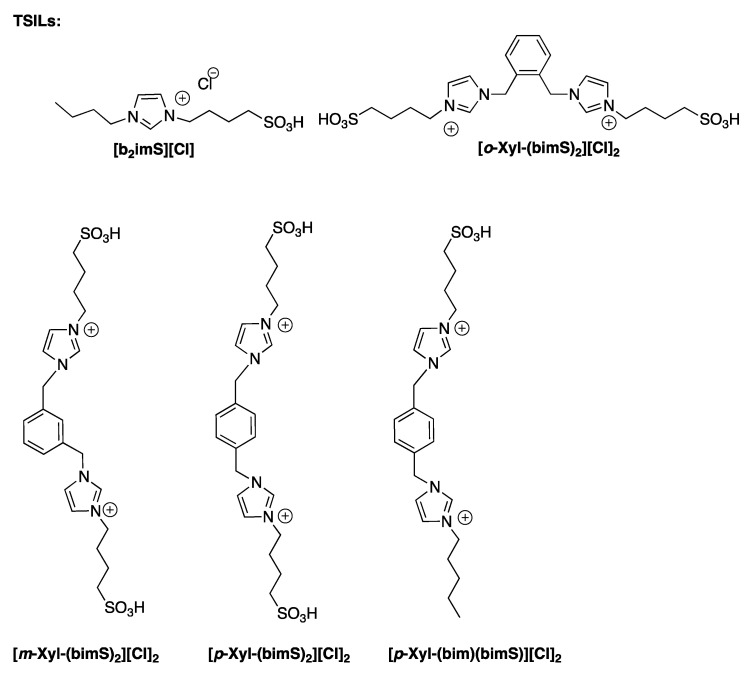
Structure of the task-specific dicationic ILs that were used as catalysts.

Fructose and sucrose dehydration have been also performed in ILs binary mixture ([bmim][Cl]_0.5_[BF_4_]_0.5_), by using dicationic TSILs, bearing different isomers of xylene as spacers (Scheme 10) [33]. For a useful comparison, the target processes were also studied in the presence of the corresponding monocationic TSIL. 

The data that were reported show that, in the used solvent system, TSDILs allowed to obtain 5-HMF from different carbohydrates, at 60 °C, in the presence of 20 mol% of catalysts.

In the case of fructose, the yield in 5-HMF decreases along the trend: [*o*-Xyl-(bimS)_2_][Cl]_2_ *~* [*m*-Xyl-(bimS)_2_][Cl]_2_ > [*p*-Xyl-(bimS)_2_][Cl]_2_ ~ [*p*-Xyl-(bim)(bimS)][Cl]_2_. The above trend was partially ascribed to the different solubility of the catalysts in the reaction mixture, as accounted for by the low solubility of [*p*-Xyl-(bimS)_2_][Cl]_2_ and [*p*-Xyl-(bim)(bimS)][Cl]_2_, that operated in non-homogeneous conditions.

On the other hand, the catalyst efficiency did not correlate with symmetry or catalyst acidity, as accounted for by the lack of correlation between the 5-HMF yield and Hammett acidity function. Rather, the efficiency of [*o*-Xyl-(bimS)_2_][Cl]_2_ was ascribed to geometrical factors and the proximity of charged heads, bearing the acidic moieties. Interestingly, monitoring the reaction mixture by ^1^H-NMR allowed to detect the formation of intermediate species that were occurring during the HMF formation. 

The use of such catalysts proved advantageous above all in the case of sucrose, as they allow to convert the disaccharide into 5-HFM, using milder reaction conditions with respect to the ones that are generally used for such a kind of substrate. Furthermore, in all cases, the solvent-catalyst system can be reused, after 5-HMF extraction with diethyl ether, for at least four cycles without a loss in yield values.

**Scheme 11:**
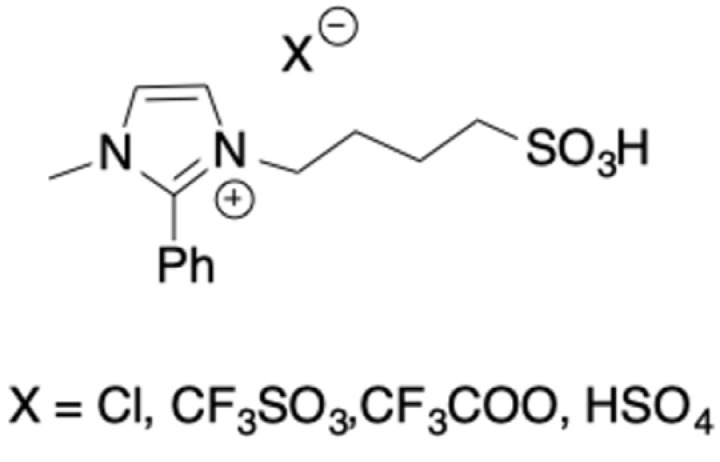
Structures of [2-PhIm-SO_3_H]-based TSILs.

Hydrophobic TSILs, bearing a phenyl ring on the C2 of the imidazolium ion, have been used to catalyze the direct conversion of microcrystalline cellulose into 5-HMF [34]. ILs that were different for the anion nature were also tested (Scheme 11).

The catalysts were fully characterized and, in particular, the TGA measurements revealed a good stability until 210 °C that made them suitable for the target process.

Conversion of microcrystalline cellulose was performed at 150 °C for 5 h, under autogenous pressure, in biphasic systems water/MIBK. The first screening that was performed by using a catalyst loading that was equal to 0.3, allowed determining the following reactivity trend: [2-PhIm-SO_3_H][CF_3_SO_3_] > [2-PhIm-SO_3_H][HSO_3_] > [2-PhIm-SO_3_H]Cl > [2-PhIm-SO_3_H][CF_3_COO]. The reactivity trend perfectly fits the acidity one, as accounted for by the Hammett acidity function and bond dissociation energies that were obtained from DTF optimized geometries.

Analysis of the different operational parameters, such as the reaction time and temperature, solvent composition, and catalyst loading, allowed to obtain 5-HMF yield that was equal to 70%, under the best experimental conditions (H_2_O/MIBK 1:10, catalyst loading: 0.3, 150 °C, 1.5 h). In particular, a solvent mixture that was rich in organic solvent allowed, also in this case, the continuous removal of 5-HMF, avoiding the occurrence of secondary degradation processes. The reaction system was recyclable with only a partial loss in yield (from 70 down to 63%) after the fourth cycle of reusing.

**Scheme 12:**
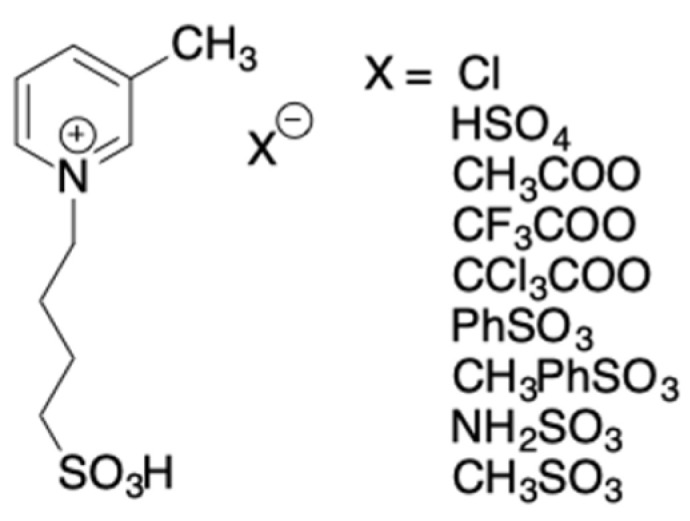
Structures of pyridinium-based TSILs.

In the framework of the use of TSILs in the dehydration of carbohydrates, also pyridinium-based ILs, bearing sulfobutyl chain on the nitrogen atom have been used (Scheme 12) [35].

They were chosen for their lower cost with respect to imidazolium ILs. These TSILs were firstly used both as solvents and catalysts, and the dehydration of fructose and glucose was carried out at 80 °C for 1 h. Analysis of the 5-HMF yields and selectivity that were obtained in the case of fructose, shed light on the very high efficiency of the *p*-toluenesulfonate salt, that allowed to obtain 5-HMF in 75% of yield with 82% of selectivity. For the other anions, the following trend was detected: [Cl^−^] > [CF_3_COO^−^] > [CH_3_COO^−^] > [CCl_3_COO^−^] > [CH_3_SO_3_^−^] > [NH_2_SO_3_^−^] > [PhSO_3_^−^] > [HSO_4_^−^], that was related to the hydrogen acceptor ability.

TSILs were also tested after conjugation with metal chlorides, such as AlCl_3_ and CrCl_3_. The metal chloride loading was firstly equal to 10% and this induced a 20% increase in the IL efficiency. Several experimental parameters were optimized, such as metal chlorides and substrate loading, reaction time, and temperature. In this case, acetate and trifluoroacetate-based ILs were tested. In the case of AlCl_3_, the best loading was equal to 3%, that gave the best yield after 1 h at 100 °C, in the presence of 10 wt% of fructose loading. However, the process was significantly improved by adding polar organic solvents to the IL, in order to increase the selectivity in 5-HMF. In particular, the use of deionized water, dimethylsulfoxide, methanol, ethanol, and isopropanol in a 1:3 ratio, was taken in consideration. Isopropanol and ethanol proved to be the best solvents in the case of fructose and glucose, that allows 94 and 53% of 5-HMF yield, respectively.

TSILs that were conjugated with metal chlorides were unable to favor cellulose conversion to 5-HMF. This was ascribed to the negative effect that was exerted by the SO_3_H group on the saccharification process. When the corresponding butyl-based ILs were used as catalysts, 54% of 5-HMF yield was collected at 150 °C, after 1 h.

In a further attempt, imidazolium-based TSILs, bearing a sulfonic acid residue in the side chain, have been applied in combination with different Lewis acids to favor the conversion of different saccharides and polysaccharides, such as sucrose, maltose, cellobiose, lactose, inulin, and cellulose into 5-HMF.

**Scheme 13:**
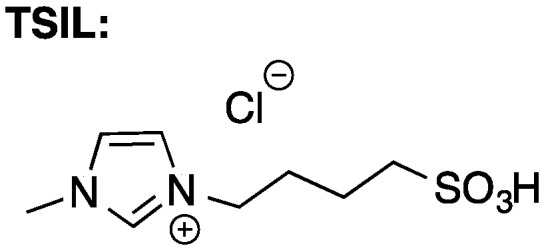
Structure of the [SO_3_H-bmim][Cl] catalyst.

The processes were performed under microwave irradiation in a butanone/water mixture (4:1). Butanone was chosen on the grounds of its renewable origin. Among the Lewis acids that were tested, the best results were collected by using the mixture of [SO_3_H-bmim]/AlCl_3_ (Scheme 13) [36].

In the case of disaccharides, with the only exception of cellobiose, the best yield values were collected after 1 min, at 170 °C. In the above conditions, the 5-HMF yield ranged from 65.1 down to 51.2%, along the order: sucrose > maltose > cellobiose > lactose. Interestingly, the above trend was related to the stability and then the rate of hydrolysis of the glucosidic bond. The solvent system could be easily recycled thanks to the change of the physical state of the mixture that was homogeneous above 150 °C, but biphasic at room temperature. This allowed to collect 5-HMF in the organic phase and the catalyst in the aqueous phase. This latter stayed active for at least three cycles.

As for polysaccharides, the behavior of inulin was similar to the ones of disaccharides and the highest yield was collected after 2 min of irradiation (59%). Differently, for cellulose, the highest yield was equal to 16.3% and the different performance of the two polysaccharides was related to the different level-off degree of polymerization that was higher for cellulose than inulin.

**Scheme 14:**
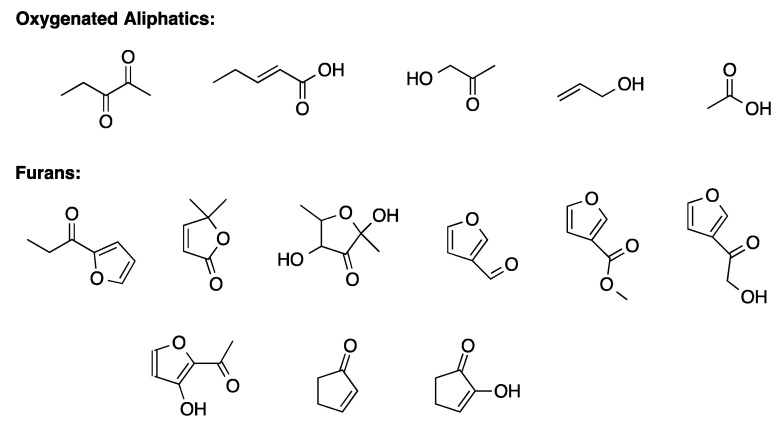
Structures of oxygenated aliphatics and furans that were produced as by-products from saccharide dehydration.

Analysis of liquid phase by GC-MS allowed identifying secondary products that were mainly furans and aliphatic oxygenated compounds (Scheme 14). 

Among furans, 5-HMF represented the 80%. The aliphatic oxygenated compounds, mainly deriving from ring opening and retroaldolization of monosaccharides, comprised 2,3-pentandione, 2-pentenoic acid, hydroxyacetone, glycolaldehyde, acetic acid, formic, and levulinic acid. Interestingly, the used irradiation method did not allow the formation of insoluble humin, while the soluble humins contained furan oligomers and unreacted sugars.

1-solfobutyl-3-methylimidazolium chloride (Scheme 13) has been also used in combination with nickel sulfate as co-catalyst, to perform the glucose conversion into 5-HMF and lactic acid in aqueous solution [19].

The process was performed under hydrothermal conditions, in the temperature range of 145–175 °C, with the aim to have a detailed kinetic investigation. This could give information about activation energy and reaction rate constants that are useful parameters in the scale-up of the process and designing the reactor. The data that were reported firstly show the synergetic action of Brønsted and Lewis acidic catalyst. In particular, the synergy factor was equal to 1.8. A maximum glucose conversion of 99.2% was achieved, with lactic acid yield that was equal to 56.33%, at 155 °C, and a maximum 5-HMF yield of 21.80%, at 175 °C. The analysis of reaction time and temperature, as well as the product distribution allowed the formulation of a detailed mechanistic hypothesis, in which the action of the Lewis catalyst proved essential in promoting the glucose-fructose isomerization, whereas the Brønsted catalyst played its role in fructose dehydration. The kinetic data that were calculated through the application of pseudo-first order model, allowed calculating activation energy and pre-exponential factor for both glucose and 5-HMF conversion into lactic acid.

**Scheme 15:**
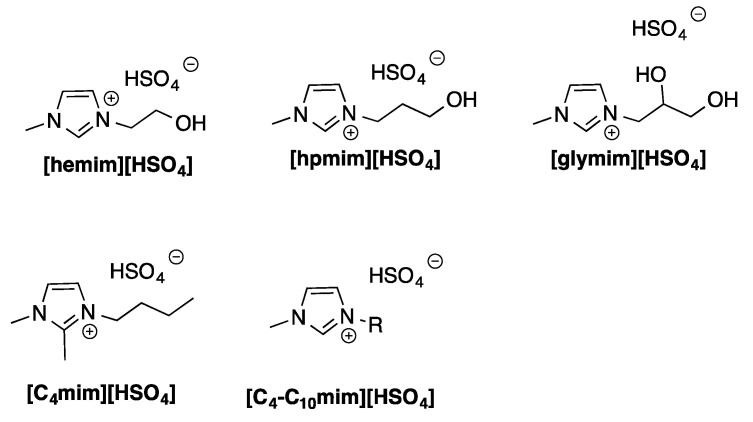
Structure of [HSO_4_^−^]-based imidazolium TSILs.

TSILs can be also featured by the presence of the catalytic function on the anion structure. On this subject, TSILs that were based on [HSO_4_^−^] anion and showing the presence of imidazolium ion bearing alkyl chain of different length (C_4_–C_10_) and, in some cases, also hydroxyl groups on the alkyl chain, were used as catalysts in water solution (Scheme 15) [37].

In this case, glucose and xylose were used as substrates. The reactions were performed at 140 °C for 4 h and in the presence of 20 wt% of H_2_SO_4_. The results that were collected prove the different effects that changing the structural parameters of TSILs has on carbohydrates conversion, as a function of the nature of the substrate. In both cases, the presence of the co-catalyst (H_2_SO_4_) exerts a positive effect. However, in the case of glucose, both the elongation of the alkyl chain or the presence of one or more hydroxyl groups on the side chain, as well as the methylation at the C2 position of the imidazolium ion, negatively affected the 5-HMF formation. In particular, the elongation of the alkyl chain caused a decrease of 5-HMF yield from 57% down to 33%. The above effects proved to be less relevant in the case of xylose and this was ascribed to its high reactivity.

5-HMF was also directly obtained from Japanese cedar, in 1-methylimidazolium hydrogenosulfate, at temperatures above 120 °C [38]. The data that were reported prove a 5-HMF yield that was equal to 7% could be obtained at 160 °C, after 30 min of treatment. This result was significantly improved by using moisture-conditioned wood (4.8% moisture content), which allowed reaching a yield of 5-HMF that was equal to 9% after 5 min of treatment. The attempt to recover 5-HMF from the reaction mixture allowed highlighting the optimum performance of the process when production of 5-HMF was carried out under vacuum steam distillation. This was the result of the combined action of the hydrophilic nature of the TSIL that, absorbing water vapor favored the hydrolysis of lignocellulosic materials, and the high boiling point and hydrophobic nature of 5-HMF.

**Scheme 16:**
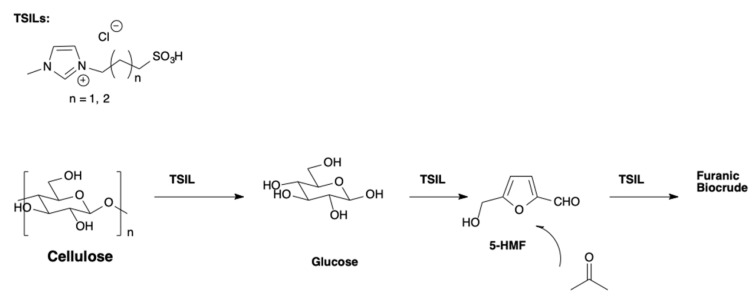
Structure of TSILs that were used as catalysts in the process of furanic biocrude formation.

1-(3-alkylsulfonic)imidazolium chlorides, differing for the alkyl chain length (Scheme 16), have been used as acidic catalysts, to produce furanic biocrude, starting from cellulose [39].

The solvothermal process, in the presence of acetone, was performed at 120 °C for 3.5 h. In the above conditions, a depolymerization, dehydration, and aldol (DDA) condensation process occurred that gave rise to the formation of 5-HMF with a yield of 57%. A detailed description of the different furanic-based side products of the reaction was given. 

The mechanism of the products formation was also rationalized. Differently from the classical pyrolysis bio-oil preparation, the proposed methodology gave a stable and well-defined C_5_–C_15_ fraction of partially oxygenated furanic compounds.

**Scheme 17:**
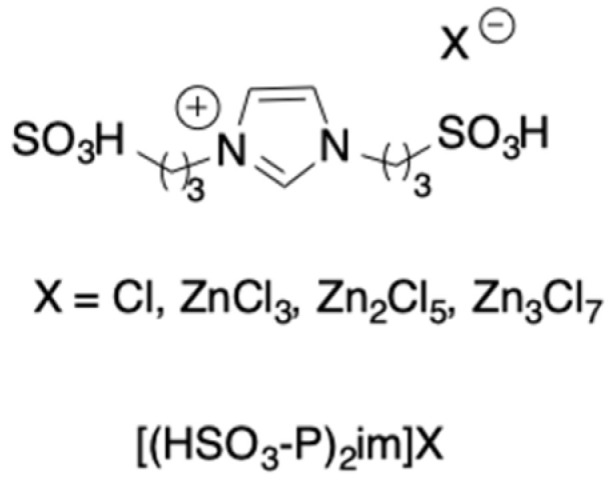
Structure of biacidic functionalized TSILs.

In a further attempt, some biacidic Brønsted and Lewis imidazolium salts (Scheme 17) were considered for the cellulose conversion to 5-HMF, and their acidities were first predicted by calculating deprotonation energy (Brønsted acidity) and LUMO energy (Lewis acidity) [40].

The results that were reported evidence how, both Brønsted and Lewis acidities increase in parallel and, in general, prove higher for disulfonic acidic ILs than for the corresponding monosulfonic ones. 

The analysis of frontier orbitals of cellulose demonstrated a drastic decrease in energy in the presence of the catalysts. Interestingly, the application of solvation model density (SMD) allowed identifying the best catalyst for the different steps, i.e., [(HSO_3_-P)_2_im][ZnCl_3_] (cellulose breakdown and fructose dehydration) and [(HSO_3_-P)_2_im][Zn_3_Cl_7_] (glucose-fructose isomerization). The results that were obtained from quantum chemical calculations were experimentally verified, carrying out cellulose conversion in DMSO at 120 °C. The first screening demonstrated the good performance of [(HSO_3_-P)_2_im][ZnCl_3_], which was able to give 36% of 5-HMF yield in 8 h. Thee investigation that was performed as a function of temperature, cellulose:catalyst ratio, and solvent amount demonstrated that 61% yield in 5-HMF could be obtained by using a substrate:catalyst ratio that was equal to 5:1, at 140 °C for 3 h, in 6 mL of DMSO. A detailed mechanistic picture was also given. 

**Scheme 18:**
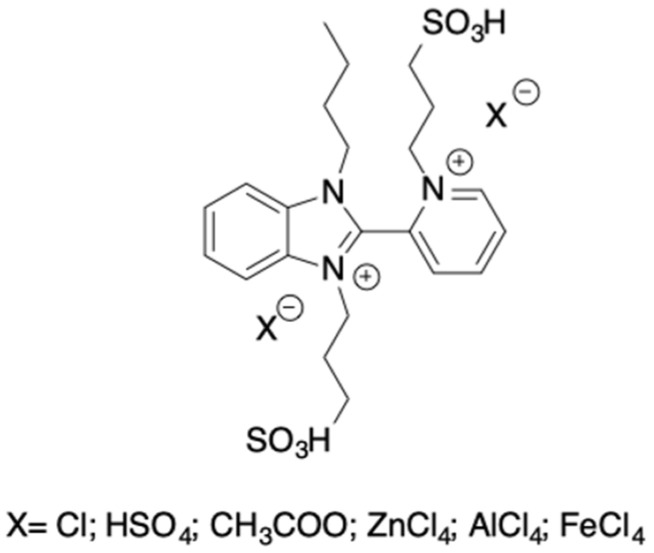
Structure of the asymmetric dicationic TSILs.

Asymmetric dicationic TSILs have been synthesized and used as catalysts, to carry out the glucose conversion into 5-HMF. Pyridinium and benzimidazolium ions were conjugated with alkyl chains bearing both sulfonate and sulfonic acid groups (Scheme 18) [41].

Anions with different basicity and anion coordination abilities, such as Cl^−^, HSO_4_^−^, and CH_3_COO^−^ were firstly tested. However, the process that was performed at 110 °C for 3 h, did not have a 5-HMF yield that was higher than 30%.

The process performance was improved when anions that were able to act as Lewis catalysts, such as ZnCl_4_^−^, FeCl_4_^−^, and AlCl_4_^−^ were used. In this case, acting in a water solution, 5-HMF yields ranging from 43 up to 71% were obtained. This was ascribed to the presence in the IL structure of both the Lewis acid site, that was able to favor glucose-fructose isomerization, and the Brønsted acid site to induce fructose dehydration to 5-HMF. The catalysts may be reused for at least five cycles without a loss in performance, and they proved to be active on different substrates, such as fructose, inulin, sucrose, and cellulose. Interestingly, a detailed mechanistic hypothesis of glucose isomerization assisted by TSILs was obtained on the grounds of DFT calculations.

Cellulose conversion has been also investigated by quantum mechanical calculations [42]. In this case, the possible use of bifunctional TSILs was taken into consideration. In particular, both ammonium and imidazolium ions bearing a -SO_3_H group on the side chain were considered, to have a Brønsted acidity function. As the salts were all designed as chloride derivatives, their Lewis acidity was modulated by their combination with different amounts of ZnCl_2_. The calculation of HOMO-LUMO gap, both in the presence and in the absence of catalyst, allowed to evaluate the energy barrier of the catalytic reaction, shedding light on the beneficial effect due to its presence. In particular, a stronger HOMO-LUMO overlap was related to higher catalytic efficiency. This kind of investigation enabled the identification of the best catalytic system, and computational prediction was fully supported by the experimental data.

**Scheme 19:**
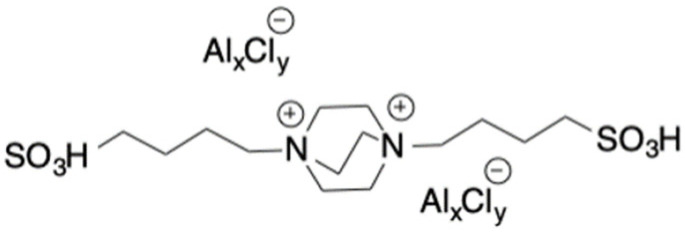
Structure of the bifunctional dicationic TSILs that were used as a catalyst.

Glucose and fructose conversion into 5-HMF has been also performed by using bifunctional dicationic DABCO-based TSILs (Scheme 19), in [emim]Cl. In this case, the Brønsted acidity function was located on the cation, whereas the Lewis function was in the anion.

In both cases, screening of operational parameter, such as the reaction temperature and time, as well as catalyst loading, was performed. In particular, in the case of glucose, the highest 5-HMF yield (30.5%) was collected at 110 °C for 1 h, by using 30 mol% of catalyst. As far as fructose was concerned, 5 mol% of catalyst was sufficient to obtain 65% of 5-HMF yield, at room temperature for 24 h. Increasing temperature to 80 °C, allowed to obtain the 99% of yield from fructose. The solvent/catalyst system was reused for at least three cycles, after the extraction of 5-HMF by using ethyl acetate/diethyl ether mixture [43].

TSILs have been also used to carry out carbohydrates conversion in conventional solvents. In this context, *N*,*N*-diethyl-1,4-phenylenediammine hydrogen sulfate [DPhDA][HSO_4_] was applied in DMSO solution, to convert glucose into 5-HMF [44]. The optimization of the operational parameters demonstrated that the highest yield (94%) could be obtained in 30 min at 160 °C, by using a catalyst loading equal to 30 mol%. In the above experimental conditions, a TOF value that was equal to 6.1 h^−1^ was calculated. In general, higher catalyst amounts induced a decrease in the catalyst performance as a consequence of the entrapment of the catalyst in the “supramolecular-like” species formed through the establishment of hydrogen bonds. Computational mechanistic studies highlighted the relevance of both basic and acidic sites of the catalyst. From an experimental point of view, this was testified by the significant decrease in yield that was detected when [DPhDA][HSO_4_] was used in combination with 18-crown-6, that was able to block the positive pole of the catalyst (NH_3_^+^), or tuff mineral that was able to establish electrostatic interactions with IL.

Fructose conversion into 5-HMF was also performed in flow conditions, by using as catalyst, the Brønsted acidic ionic liquid [bmimSO_3_H][HSO_4_] [45]. The process was performed at 130 °C and the best yield in 5-HMF (55%) was obtained in 45 s of residence time. The above result proved to be valuable, considering that *p*-TsOH, allowed to obtain the same yield, at the same temperature, only after 30 min.

**Scheme 20:**
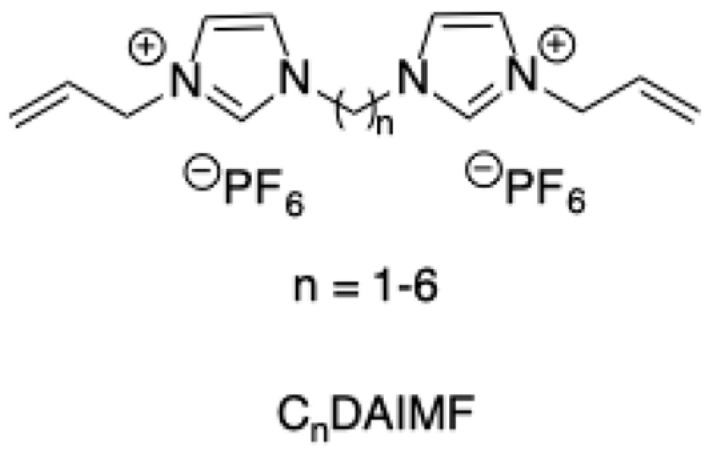
Structure dialkyl-imidazolium salts.

In a further attempt, dialkyl-imidazolium salts bearing the [PF_6_^−^] anion and differing for the alkyl spacer between the charged heads (Scheme 20), have been prepared and tested as catalysts in the conversion of fructose to 5-HMF [46].

The process was carried out under microwave irradiation in water or DMSO solution. The results that were reported evidence the role that is played by the nature of the imidazolium salts, since dicationic ILs allowed the obtainment of a higher yield with respect to the corresponding monoimidazolium salts. As for dialkyl-imidazolium salts, better results were collected using [C_2_DAIM][PF_6_], indicating that the ethyl derivative was the most effective catalyst. This catalytic activity was higher in the DMSO than in the water solution, and higher yields in 5-HMF were collected in the presence of [PF_6_^−^] anion compared with the Br- anion. The highest yield in 5-HMF was equal to 75%, obtained at 195 °C, and was comparable to the one that was achieved in the presence of solid acidic catalysts.

## 4. Heterogeneous Catalysis

A widely used strategy for the obtainment of 5-HMF from carbohydrates and biomasses employs heterogeneous catalysts [47]. Heterogeneous catalysis provides several advantages over homogeneous catalysis in terms of easier separation and recycling of the catalyst, as well as lower corrosivity if compared with strong acids that are conventionally used for sugar conversion into 5-HMF. Among the various classes of heterogeneous catalysts that have been recently employed to obtain 5-HMF, zeolites emerge with a prominent role [48].

**Scheme 21:**
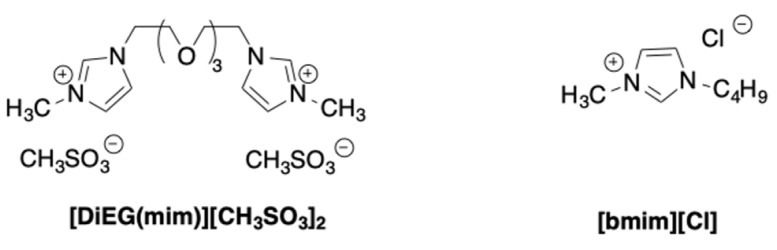
ILs used as templating agent and solvent.

Zeolites are aluminosilicates with a crystalline microporous framework, and can possess both Brønsted and Lewis acidity, which in turn depend on their Si/Al ratio. The presence of a framework with defined dimensions allows shape selectivity and molecular traffic control when the reactants enter a type of pore and the products are released from a different type of pore. Finally, native zeolites can be modified to best suit the catalyzed process, mostly by varying the acidity and density of catalytic sites. In the context of zeolite-promoted dehydration of fructose in IL, a ZSM-5 zeolite was modified by performing its synthesis in the presence of the dicationic IL [DiEG(mim)][CH_3_SO_3_]_2_ as a templating agent (Scheme 21), using the resulting material as catalyst [49].

Optimization of the reaction conditions showed that the best yield in 5-HMF was 67%, in 90 min, at 110 °C in [bmim][Cl]. Notably, the template synthesis altered the porosity of the pristine zeolite, enhancing the catalytic efficiency and the reaction was conducted at a gram scale.

Zeolites that were modified with metal ions have been reported for the dehydration of carbohydrates into 5-HMF. In this context, calcined USY- and Beta zeolites that were modified with Cr(III) ions, were shown to efficiently promote the obtainment of 5-HMF from glucose and cellulose [50], in [bmim][Cl] as a solvent, with yields of 59% and 34%, respectively, after 1 h, at 130 °C. However, significant leaching of chromium in IL occurred, which prevented recycling of the catalyst.

In a recent example, a zeolitic multifunctional catalyst was obtained by first doping HY zeolite with Ru(III) and then functionalizing the ensuing material with SO_3_H-groups [51]. The catalyst proved efficient in promoting the formation of 5-HMF from microcrystalline cellulose. In particular, the reaction was carried out in the IL [emim][Cl], in the presence of methylisobutyl ketone (MIBK) as an extracting phase, obtaining a yield of 48% at the relatively low temperature of 120 °C. The result was attributed to the cooperative action of the strongly acidic groups on the zeolite and the ability of the Ru centers, which behave as a plasmonic species. As a result, the catalytic activity is enhanced by a surface plasmon resonance effect (SPR), in which light is harvested to generate a local thermal effect. 

**Scheme 22:**
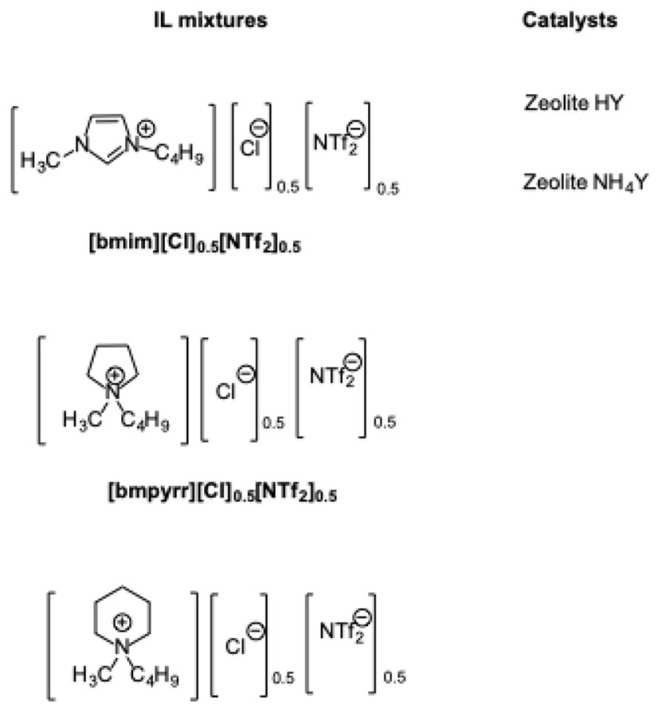
Conversion of carbohydrates into 5-HMF, IL mixtures that were used as solvents and the catalyst that was employed.

Native zeolites can also efficiently catalyze the dehydration of carbohydrates into 5-HMF. In this regard, zeolites NH_4_Y and HY proved efficient for the conversion of fructose, glucose, and sucrose, in IL mixtures as solvent media (Scheme 22) [52].

In particular, the solvent media that were used were mixtures of aromatic as well aliphatic ILs. The optimization of reaction conditions showed that the best catalyst was the HY zeolite, due to its higher acidity. Moreover, the extent of proton exchange between zeolite and ILs depended on the composition of the mixtures, and was more pronounced in the [bmpyrr][Cl]_0.5_[NTf_2_]_0.5_ mixture. Under the optimal experimental conditions, the yield in 5-HMF amounted to 73% in 5 h at 80 °C and 62% in 3 h at 120 °C from fructose and sucrose, respectively. Conversely, from the less reactive substrate, glucose, the yield amounted to 38% in 4 hours at 120 °C. It is worth noting that these results were obtained at substantially lower temperatures than the ones that were described previously. Notably, in the case of glucose and sucrose, the best results were obtained in the aliphatic IL mixtures, which are widely regarded as less toxic than their aromatic counterpart. Finally, the dehydration of fructose could be conducted under probe sonication, obtaining a yield of 71% in only 0.5 h, as opposed with the 5 h that was required in silent condition, coupled with a remarkable reduction in the reaction temperature, which was equal to 40 °C.

Minerals that are doped with metal ions to enhance Lewis acidity can serve also as catalysts for dehydration of carbohydrates using ILs as solvents. In this regard, Ju et al. described an Al- and Sn-modified hydroxyapatite as an efficient catalyst for dehydration not only of monosaccharides, but also of disaccharides as sucrose, maltose, and cellobiose, as well as a polysaccharides such as starch [20].

**Scheme 23:**
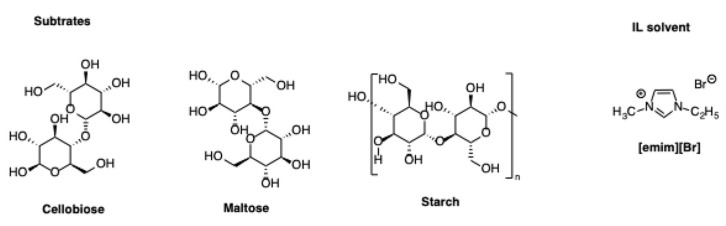
Di- and polysaccharide substrates and IL solvent that were used for carbohydrate dehydration to 5-HMF.

The reactions were carried out in the IL [emim][Br] as solvent (Scheme 23).

Incorporating the metal center in hydroxyapatite, imparted high Lewis- and moderate Brønsted acidity to the catalyst, which proved favorable for the process that was studied. In particular, under optimized conditions, the yield of 5-HMF that was obtained from glucose amounted to 70% in 3 h at 130 °C, with a glucose loading of 10 wt%. Increasing the initial glucose loading induced a reduction in the yield due to the occurrence of side reactions. However, a good yield of 47% was still observed when increasing the initial amount of glucose up to 40 wt%. Good yields were also obtained from sucrose, cellobiose, and maltose, amounting to 74%, 61%, and 63%, respectively. Notably, a much more recalcitrant substrate such as starch, gave 5-HMF in 47% yield. Finally, a mechanistic investigation revealed that Brønsted acidic sites within the catalyst promoted the depolymerization of starch, while the intermediate isomerization step from glucose to fructose was mainly promoted by the Lewis acidic sites. Furthermore, an assistance of the IL [emim][Br] in the final dehydration step, leading from fructose to 5-HMF was hypothesized. Along the same line, Cr(III)-exchanged montmorillonite and bentonite were employed as catalysts for the dehydration of glucose in the IL [bmim][Cl] [53]. Although a yield in 5-HMF of 45% at 140 °C and 63% at 150 °C, were observed in the presence of Cr-montmorillonite and bentonite, respectively, metal ion leaching in the IL occurred during the reaction and the catalyst was not recyclable.

Given that the dehydration of carbohydrates into 5-HMF is catalyzed by Lewis acids, metal oxide have also proved effective catalysts. In particular, by treating aluminum oxide with suitable solutions of NaOH and HCl, it is possible to adjust the Lewis and Brønsted acidity of the ensuing material, to reach a high Lewis acidity that is coupled with a moderate Brønsted acidity [54]. These materials acted as a catalyst for the glucose dehydration in the IL [emim][Br] as solvent, and the best performing material allowed obtaining the relatively good yield of 50% (3 h, 140 °C), from a high concentration glucose (10 wt%). Metal oxides have also proven to be efficient in promoting the conversion of polysaccharides into 5-HMF. 

In this context, a boehmite (γ-AlOOH) catalyst was employed for the conversion of both glucose- and fructose-based polysaccharides such as cellulose and inulin, respectively [55]. The reactions were performed in [bmim][Cl], with DMSO and water as co-solvents. The yields in 5-HMF from cellulose and inulin amounted to 58% and 70%, respectively, while starch afforded a yield of 63%, after 2 h at 160 °C. The recyclability of the catalyst was tested using cellulose as a substrate, finding no obvious loss in performance over five consecutive runs. As previously seen when discussing the use of zeolite-based catalysts, doping metal oxides with Sn(IV) can enhance the Lewis acidity to allow isomerization of glucose to fructose as an intermediate step in the obtainment of 5-HMF. In this regard, Sn-doped Al_2_O_3_ was employed as a catalyst for the conversion of glucose in high concentration, 35 wt%, in 5-HMF, using [emim][Br] as solvent [56]. Conducting the reaction at 140 °C for 1 h afforded almost quantitative conversion of glucose and a yield in 5-HMF of 51%. Furthermore, prolonging the reaction time to 3 h or 4 h, good yields in 5-HMF were obtained from disaccharides as sucrose, cellobiose, and maltose, amounting to 63%, 43%, and 44%, respectively. Among the polysaccharides, a good 39% yield was obtained from starch, although practically no 5-HMF was obtained starting from cellulose. This result was ascribed to the lower solubility of cellulose in the IL.

Among the heterogeneous catalysts that were employed to promote the production of 5-HMF from carbohydrates, suitably activated carbons have recently gained interest. Activated carbon is a form of purified charcoal with small low volume pores and high surface area [57]. Besides their catalytic ability, an advantage of activated carbon from the sustainability point of view, is that it can be obtained from the thermal treatment of biomasses and even waste. 

In this regard, activated carbon that was previously treated with sulfuric acid was employed for the obtainment of 5-HMF from forestry biomass of *Acacia nilotica*, in the IL [bmim][Cl] [58]. In particular, the wood was first pretreated and dissolved in the IL, then the catalyst was added. The reaction was performed at 120 °C for 60 min, which represent remarkably mild conditions for this process, which afforded a notable yield in 5-HMF of 58%.

A similar carbon material than has been increasingly used as catalyst for biomass upgrading, is biochar, which is obtained by thermochemical degradation of lignocellulosic biomass [59]. Biochar is inexpensive, environmentally friendly, and easily produced. Moreover, biochars with high surface area can be conveniently functionalized or activated to enhance their catalytic ability [60]. Along this line, boron-doped biochars were employed as heterogenous catalysts for the one-pot conversion of corncob, rice straw, and wheat straw 5-HMF, using the IL [emim][Cl] as solvent, in the presence of boric acid [61]. This latter provided Brønsted acidity, while the modified biochar mainly acted as a Lewis acid. The highest yields were obtained from corncob as a substrate, which reached 21% (140 °C, 2 h), whereas substantially lower yields were observed from the other biomasses. Kinetic analysis revealed a synergistic role of the two catalysts, biochar and boric acid.

Carbonaceous microspheres that were obtained by hydrothermal carbonization of yeast cells were functionalized with sulfonic groups (-SO_3_H) and employed as catalysts for the conversion of fructose and sucrose, as well as the fructose-based polysaccharide inulin, in [bmim][Cl] [62]. Under optimized conditions at 80 °C, yields of 83%, 45%, and 59% were reported from fructose, sucrose, and inulin, respectively. The catalyst could be recycled eight times only in the case of fructose, whereas for the conversion of sucrose and inulin, a drop in performance occurred after the first cycle. Using a similar approach, a carbonaceous catalyst was prepared from carbonization of cotton gin trash, which was subsequently subjected to chlorosulfonation [63]. Fructose dehydration was carried out at 80 °C in [bmim][Cl] with acetone as co-solvent for 20 min, affording a yield of 71%. Moreover, the catalyst could be reused maintaining performance over six cycles. 

Employing a similar approach, catalysts for the dehydration of fructose were obtained from hydrothermal carbonization of soft wood pulp, which was then treated with sulfuric acid to induce sulfonation of the aromatic rings [64]. This acidic catalyst was then used to promote the dehydration of fructose in 5-HMF, in the IL [bmim][Cl], in the presence of MIBK. Notably, the reaction in the IL afforded 5-HMF with negligible formation of side-products, whereas when the reaction was carried out with the same catalyst in a conventional solvent such as DMSO, a significant reduction in selectivity occurred. Based on experiments that were conducted under different conditions, a quantitative model was developed, expressing a yield in 5-HMF as a function of the reaction time, temperature, and catalyst loading as variables. This model suggested that the optimum reaction conditions would be 112 °C, 24 min, and 5 wt% of catalyst loading, with a good match between the predicted and actual yield which amounted to 99% and 96%, respectively. Upon recycling the catalyst, a significant drop in the yield occurred, due to obstruction of the catalyst pores by humins. However, the efficiency of the catalyst could be restored by washing with acetone prior to reusing it. 

**Scheme 24:**
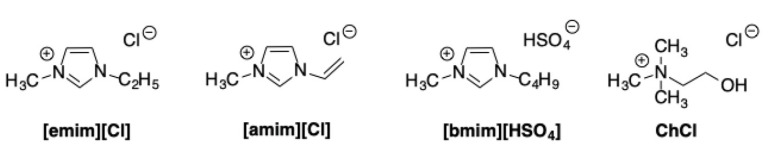
Imidazolium ILs and choline chloride that were used in the modified peanut shell catalyzed conversion of carbohydrates into 5-HMF.

Finally, a heterogeneous catalyst for carbohydrate conversion into 5-HMF was obtained by impregnation of hydrothermally carbonized peanut shells with sulfuric acid [65]. In this case, the reaction was conducted in imidazolium ILs such as [amim][Cl], [emim][Cl], and [bmim][HSO_4_], in the presence of choline chloride (ChCl) and DMSO, Scheme 24.

It was found that the selectivity greatly improved in the presence of both DMSO and ChCl, since they inhibited the degradation of the 5-HMF into side products such as humins or levulinic acid. However, this system showed a somewhat limited scope with a good yield from glucose, 55% in [bmim][HSO_4_]-ChCl-DMSO, at 130 °C in 2 h, whereas negligible yields in 5-HMF were observed from cellulose or other lignocellulosic biomass such as reed, stalk, wheat straw, and peanut shell waste. Notably, when fructose was used as substrate, the presence of ILs appeared detrimental and the yields that were obtained were inferior to the one that was detected in ChCl-DMSO.

Lewis acidic chromium chlorides, CrCl_2_ and CrCl_3_, were among the earliest catalysts that were used for the conversion of carbohydrates into 5-HMF, and were especially effective in promoting the dehydration of glucose, although their use raises some issues from the standpoint of sustainability, such as their toxicity and the corrosiveness of the reaction media in which they are dissolved. However, they can find application promoting the obtainment of 5-HMF from more recalcitrant feedstock, such as cellulose waste, deriving from materials with different polymerization degrees. In this regard, it was found that CrCl_3_ in the presence of AlCl_3_ as a co-catalyst, is efficient in promoting 5-HMF formation in good yields from materials such as cotton or filter paper [66]. In particular, the reactions were conducted at 120 °C for 3 h in [bmim][Cl], affording comparable yields from microcrystalline cellulose, cotton, and filter paper alike, amounting to 58%, 59%, and 54%, respectively. Mechanistic investigation suggested that AlCl_3_ is mainly effective in promoting the breakdown of cellulose into glucose, whereas CrCl_3_ acted as a catalyst for the conversion of the latter into 5-HMF.

Ionic exchange resins, particularly the acidic ones, are a class of polymeric materials that have, in many instances, proved effective catalysts for the conversion of carbohydrates into 5-HMF. On this topic, the acidic ion exchange resin D001-cc was used as a catalyst for the obtainment of 5-HMF from sugarcane bagasse [67]. In this case, the biomass was first pretreated under probe sonication in the IL [bmim][CH_3_COO], to reduce its degree of crystallinity. Then, the treated biomass was recovered by precipitation with water, mixed with the IL solvent, and the catalyst and the reaction was conducted under microwave irradiation. The best results were obtained by irradiating at 140 °C for 25 min in the IL [bmim][CH_3_COO], with a yield of 66%. Notably, in this IL the yield was higher than the ones that were obtained in [bmim][Cl] as well as in the conventional solvents DMSO and water, due to higher solubilizing power of [bmim][CH_3_COO]. Using the same approach and catalyst, allowed the same group to achieve good yields of 5-HMF from apples and citrus fruit waste [68]. The optimization of the reaction conditions showed that the best results were obtained at 160 °C, after 40 min in the IL [bmim][Cl], with a yield of 5-HMF of 45%. Also in this case, ultrasonic pretreatment of the sugary food waste was mandatory to achieve good conversion to 5-HMF. Finally, kinetic analysis demonstrated that an increase in temperature mainly favored the hydrolysis of cellulose over the other steps.

A closely related type of acidic polymeric catalyst was obtained by cross-linking polyvinylalcohol (PVA) in the presence of solfosuccinic acid, which resulted in functionalization of the polymer with sulfonic acid groups [69]. By adjusting the cross-linker concentration, it was possible to tune the properties such as the thermal stability, crystallinity, and density of the catalytically active groups, and, in turn, the catalytic efficiency. Such solid catalysts were employed for the dehydration of fructose, glucose, and sucrose into 5-HMF, in a [bmim][Cl]/water (5:1, *w*:*w*) solvent mixture, in the presence of MIBK as extracting phase. Under optimized conditions, the reactions were performed at 117 °C, obtaining a 99% yield after 40 min from fructose. Moderate yields were observed from glucose and sucrose, amounting to 25% after 120 °C and 64% after 60 min, respectively. In all cases, the catalysts performed better in the IL-water mixture compared with a purely aqueous medium. The best performing catalyst showed good recyclability, with the yield keeping constant over five consecutive runs.

A different class of solid acidic catalysts that were employed in biomass upgrading, are represented by heteropolyacids [70,71]. These latter are transition metal oxide clusters, that are endowed with strong Brønsted acidity. In this regard, a hafnium-based heteropolyacid that was modified by treatment with sulfuric acid (VNU-11-SO_4_) was employed to promote the conversion of monosaccharides such as fructose and glucose into 5-HMF, in the IL [emim][Cl] [72]. The reactions were carried out at 110 °C, and the highest yields were obtained were 86% and 28% from fructose and glucose, respectively. The performance of the catalyst worsened in a conventional solvent such as DMSO as well as in deep eutectic solvents. Leaching of hafnium was limited, 2 ppm, however only a moderate recyclability was found, with yield slightly dropping after three cycles.

Molecular sieves can be modified by the ionothermal method, to include catalytically active metal centers. In this regard, molecular sieves that were modified with trace Cu(II) (Cu-APO-5) were described as catalysts for the conversion of fructose to 5-HMF [73]. Performing the reaction in [bmim][Br] afforded up to 94% yield, at 100 °C in only 3 min. The catalyst could also be reused up to five runs with no obvious drop in performance.

As previously mentioned, a key step to enable efficient upgrading of cellulosic biomass into 5-HMF, is the isomerization of glucose into the much more reactive fructose. This is because glucose and glucose-based polysaccharides are by far the most abundant fraction of cellulosic biomass. It is, therefore, reasonable to use enzymes such as glucose isomerase (GI) to promote this process. Enzymes in general provide the advantages of being specific, selective, and proceeding under mild reaction conditions with very low environmental impact [74]. Consequently, coupling enzymatic catalysis for the isomerization step to a chemical one for the fructose dehydration step is a viable strategy to achieve the efficient production of 5-HMF from biomass [75].

In the context of chemo-enzymatic conversion of carbohydrates into 5-HMF, both immobilized GI and GI from crude cell extract of *Streptomyces coelicolor,* were coupled to the ion exchange resin Amberlyst 15, for the sequential conversion of glucose [76]. In particular, the isomerization step was carried out in water at 60 °C, while the dehydration step occurred in H_2_O/[bmim][Cl]_0.5_[BF_4_]_0.5_ (1:3, *w*:*w*) ternary mixture, at 100 °C. In the presence of commercial GI, an overall 40% yield in 5-HMF after 4.5 h was observed, whereas employing the cell-extract-derived GI afforded a yield of 50% after 24 h for isomerization and 1 h for the dehydration reaction. Moreover, both isomerization and dehydration steps benefited from US activation, affording yields that were similar to the ones that were obtained in silent conditions but at lower temperatures and shorter reaction times. In particular, US activation allowed the dehydration step to proceed at 50 °C instead of 100 °C with no reduction in yield. 

To sum up, we report below Table 1, containing a selection of the works that were covered in this review, describing the catalysts, reaction conditions, and yields for each substrate.

## 5. Conclusions

The urgent need to reduce the dependence of the modern society on fossil fuels has induced a growing interest in studying catalytic processes that, starting from biomass, could allow to obtain chemical intermediates of industrial value. In this context a key role is played by carbohydrates conversion into 5-HMF. This process has been widely studied in conventional solvents and more recently in ILs solution.

Analysis of the most recent literature sheds light on the advantages in using these solvents for the target process. Indeed, ILs frequently allow the obtainment of 5-HMF with a higher yield, selectivity, and under milder reaction conditions with respect to conventional solvents. The selectivity increase is considered the result of the stabilizing effect that, above all, IL anions are able to exert on 5-HMF, avoiding secondary processes.

ILs, thanks to their low miscibility with conventional solvents, make the 5-HMF recovery easier, allowing the reuse of the solvent/catalyst system. This aspect, which is always important from an environmental point of view, becomes more significant as far as the use of highly acidic catalysts is concerned.

A strong point in the ILs use for the obtainment of 5-HMF is represented by the possibility to convert these simple salts in species, such as TSILs, that play the dual role of solvent and catalyst, further decreasing the number of species that are involved in the process and consequently, the environmental impact.

Looking at Table 1, it can be observed that heterogeneous catalysts appear more suitable than homogeneous ones for the conversion of polysaccharides and more recalcitrant lignocellulosic biomass. Consequently, future work in the field should focus on this kind of catalyst to enlarge the gamut of lignocellulosic biomass for the obtainment of 5-HMF.

On the other hand, analysis of the most recent literature demonstrates that, although successful, the scope of homogeneous catalysts in ILs in this field is mainly related to the conversion of mono-and disaccharides, evidencing the need that, in the near future, more and more papers will deal with the obtainment of 5-HMF from raw polymeric materials.

Another important issue to be addressed is the recyclability of ILs. In the vast majority of the work that is presented, ILs are recycled for no more of five to six cycles. Therefore, future research should consider the development and use of more recyclable ILs for the obtainment of 5-HMF.

Lastly, the conversion of lignocellulosic biomass into 5-HMF in ILs appears to be a promising field for the application of non-conventional means of activation, such as ultrasounds or microwave irradiation, that are able to allow conversion under lower temperatures and shorter reactions times. Furthermore, more examples of 5-HMF formation from the sources that are carried out under continuous flow conditions are increasingly emerging. Thus, future work should also investigate in this direction.

## Figures and Tables

**Table 1:** Catalysts, reaction conditions, and yields for the selected transformations of carbohydrates and biomass into 5-HMF.

Catalyst	Substrate	Time	Temperature (°C)	Yield in 5-HMF (%)
Homogeneous Catalysis				
HCl [26]	Fructose	1 h	120	90
Tricationic ILs [30]	Fructose	3 h	120	93
Acid functionalized ammonium ILs [26]	Fructose	0.5 h	120	87
CrCl_3_ [29]	Glucose	2 h	120	62
Tricationic ILs [30]	Glucose	3 h	140	51
Protic imidazolium IL [38]	Japanese Cedar	5 min	160	9
HCl [26]	Sucrose	2 h	110	80
Tricationic ILs [30]	Sucrose	3 h	120	72
Acid functionalized imidazolium ILs/AlCl_3_ [36]	Sucrose	1 min	170	65
**Heterogeneous Catalysis**				
Sulfonated-activated carbon [58]	*Acacia nilotica* biomass	1 h	120	58
Sn-modified hydroxyapatite [53]	Cellobiose	3 h	130	61
Bohemite [55]	Cellulose	2 h	160	58
CrCl_3_/AlCl_3_ [66]	Cellulose	3 h	120	58
B-doped biochar [61]	Corncob	2 h	140	21
CrCl_3_/AlCl_3_ [66]	Cotton	3 h	120	66
HY zeolite [52]	Fructose	30 min	40	71
Cu-doped molecular sieves [73]	Fructose	3 min	100	95
Sn-modified hydroxyapatite [53]	Glucose	3 h	130	70
Bohemite [55]	Inulin	2 h	160	70
Sn-modified hydroxyapatite [53]	Maltose	3 h	130	63
Sn-modified hydroxyapatite [53]	Starch	3 h	130	47
Sn-doped Al_2_O_3_ [56]	Sucrose	4 h	140	63
D001-cc ion exchange resin [67]	Sugarcane bagasse	25 min	140	66

## Data Availability

Not applicable.
